# SecProCT: In Silico Prediction of Human Secretory Proteins Based on Capsule Network and Transformer

**DOI:** 10.3390/ijms22169054

**Published:** 2021-08-22

**Authors:** Wei Du, Xuan Zhao, Yu Sun, Lei Zheng, Ying Li, Yu Zhang

**Affiliations:** Key Laboratory of Symbol Computation and Knowledge Engineering of the Ministry of Education, College of Computer Science and Technology, Jilin University, Changchun 130012, China; weidu@jlu.edu.cn (W.D.); zhaoxuan20@mails.jlu.edu.cn (X.Z.); ysun18@mails.jlu.edu.cn (Y.S.); zhenglei2119@mails.jlu.edu.cn (L.Z.); liying@jlu.edu.cn (Y.L.)

**Keywords:** secretory protein, deep learning, convolutional neural network, capsule network, transformer

## Abstract

Identifying secretory proteins from blood, saliva or other body fluids has become an effective method of diagnosing diseases. Existing secretory protein prediction methods are mainly based on conventional machine learning algorithms and are highly dependent on the feature set from the protein. In this article, we propose a deep learning model based on the capsule network and transformer architecture, SecProCT, to predict secretory proteins using only amino acid sequences. The proposed model was validated using cross-validation and achieved 0.921 and 0.892 accuracy for predicting blood-secretory proteins and saliva-secretory proteins, respectively. Meanwhile, the proposed model was validated on an independent test set and achieved 0.917 and 0.905 accuracy for predicting blood-secretory proteins and saliva-secretory proteins, respectively, which are better than conventional machine learning methods and other deep learning methods for biological sequence analysis. The main contributions of this article are as follows: (1) a deep learning model based on a capsule network and transformer architecture is proposed for predicting secretory proteins. The results of this model are better than the those of existing conventional machine learning methods and deep learning methods for biological sequence analysis; (2) only amino acid sequences are used in the proposed model, which overcomes the high dependence of existing methods on the annotated protein features; (3) the proposed model can accurately predict most experimentally verified secretory proteins and cancer protein biomarkers in blood and saliva.

## 1. Introduction

Human secretory proteins can enter the blood, saliva or other body fluids through various complex secretory pathways and can be used as protein markers for the detection of blood, saliva or other body fluids [1]. The complex blood circulation system of the human body has many biomarkers that can indicate physiological conditions and disease conditions. Therefore, most current studies on biomarkers in body fluids use blood as the main research object [2]. Similar to other human body fluids, saliva is rich in biomolecules secreted from salivary glands or leaked from nearby tissues [3]. In addition, biomolecules can also be released into the blood circulatory system through various organs far away from the salivary glands into the human body and then be secreted into saliva [4]. Therefore, the biomolecules in saliva can reflect the health of specific organs to a certain extent, including organs both near and far away from the salivary glands.

At present, there have been many studies conducted to predict which proteins are located on the cell surface or secreted into the extracellular environment [5,6,7]. The methods proposed in these studies are mostly based on annotation information such as the amino acid compositions of protein, protein domains and protein functions. These existing methods play a very important role in the diagnosis of early cancer and other diseases, and to a certain extent, they solve the problem of predicting human secretory proteins. However, most of these methods are based on conventional machine learning algorithms. The prediction process in most machine learning algorithms can be described in two steps: first select the features from the constructed feature set, and then train the machine learning prediction algorithm using the divided training set [8]. However, incomplete features or selection bias are very common in the feature selection process, which may have a negative impact on the final prediction result [9].

The existing studies are shown in Table 1, which are mostly based on annotation information such as the amino acid compositions of protein, protein domains and protein functions. Cui et al. proposed a prediction model based on machine learning algorithms using data mining techniques to predict which human proteins can be secreted into the blood [10]. Liu et al. proposed a method for predicting blood-secretory proteins based on manifold ordering [11]. Wang et al. presented a novel computational method for predicting salivary proteins that come from the circulation based on a set of physiochemical and sequence features [12]. Sun et al. proposed a computational method for predicting secretory proteins in human saliva [8]. Zhang et al. proposed a predictor based on sequence features for the high-throughput and accurate identification of mammalian secretory proteins [13]. Recently, Zhang et al. also proposed a prediction method for blood-secretory proteins based on the optimisation of the discrete firefly algorithm [14].

In this article, we propose an end-to-end prediction model based on a deep learning framework, which is mainly comprised of a capsule network and transformer architecture, to predict secretory proteins using only amino acid sequences. Our model can accurately identify human blood and salivary secretory proteins only based on sequence information. How to effectively represent the protein data for the input of the neural network is another key issue that urgently needs to be solved. The data input representation of the network directly determines the effectiveness of a series of settings, such as the network structure, loss function, and hyperparameters, and determines the upper limit of the final predictive performance of the model. Therefore, in the pre-treatment process, our main task is to design effective protein sequence encoding. A simple and widely used protein sequence encoding method is one-of-*K* encoding. The data for the protein encoding consist of a matrix of *N* rows and *K* columns, where *K* is usually the number of amino acid types and *N* is the length of the input protein sequence [9]. In the matrix, each column corresponds to a type of amino acid, and each row represents the position in the protein sequence. The *K*-dimensional vector represented by the row has a value of 1 at the index where the corresponding amino acid appears and has a value of 0 at the other positions. However, one-of-*K* encoding does not consider the evolutionary relationships among different proteins. Therefore, in this article, we used PSI-BLAST [15] to perform sequence alignment in the “Uniref50” database [16] to obtain evolutionary profile information for protein sequence encoding. To solve the problem of unbalanced data, the bagging ensemble learning method is applied during the training process. Then, the PSSM of the training data is input into the proposed model to train the model parameters.

The proposed model achieves high accuracies using cross-validation (0.921 and 0.892 for predicting blood-secretory proteins and saliva-secretory proteins, respectively) and on an independent test set (0.917 and 0.905 for predicting blood-secretory proteins and saliva-secretory proteins, respectively), thus outperforming existing methods based on conventional machine learning algorithms. By comparing the results of the proposed model with experimentally detected blood-secretory proteins and saliva-secretory proteins, our model can achieve true positive rates of 0.909 and 0.935, respectively. By comparing the results of the proposed model with known cancer biomarkers in blood and saliva, our model can achieve true positive rates of 0.884 and 0.946, respectively. A web server for predicting secretory proteins was developed, which can be accessed via the following website: http://www.csbg-jlu.info/SecProCT/, accessed on 21 August 2021. We believe that the proposed model and web server are useful for biomedical researchers who are interested in finding protein biomarkers in blood and saliva, especially when they have candidate proteins obtained from transcriptome or proteome data.

The main contributions of this article are as follows: (1) a deep learning model based on a capsule network and transformer architecture is proposed for predicting secretory proteins. The results of the model are better than the existing conventional machine learning methods and deep learning methods for biological sequence analysis; (2) only amino acid sequences are used in the proposed model, which overcomes the high dependence of existing methods on the annotated protein features; (3) the proposed model can accurately predict most experimentally verified secretory proteins and cancer protein biomarkers in blood and saliva.

The rest of this paper is organised as follows: the Related Work Section surveys the related work for predicting secretory protein. The Materials and Methods Section presents the datasets and technical details of the proposed method. The Results Section gives the performance evaluation. Finally, the Conclusions Section concludes this paper and pinpoints future works.

## 2. Results

### 2.1. Evaluating the Performance of the Binary Classification

To evaluate the performance of the proposed model on the test set, we used bagging ensemble learning for 10 iterations to train our proposed model. For blood-secretory proteins, 380 blood-secretory proteins and 380 non-blood-secretory proteins were used to train the model in each iteration, and the model training was evaluated on an independent test set consisting of 106 blood-secretory proteins and 100 non-blood-secretory proteins. For saliva-secretory proteins, 350 saliva-secretory proteins and 350 non-salivary secretory proteins were used to train the model in each iteration, and the model training was then evaluated on an independent test set consisting of 100 salivary secretory proteins and 100 non-salivary secretory proteins. Then, the average of the results of 10 iterations was calculated as the final prediction result. The performance metrics of SecProCT and the other methods are shown in Table 2 and Table 3. On the independent test set, the average accuracy, sensitivity, specificity, MCC and AUC for predicting blood-secretory proteins and saliva-secretory proteins were 0.917, 0.906, 0.930, 0.835, 0.967 and 0.909, 0.898, 0.919, 0.817, 0.956, respectively.

The performance metrics of SecProCT and the other methods are shown in Table 2 and Table 3. Among them, the SVM methods for predicting blood-secretory protein and saliva-secretory protein were proposed by Cui et al. [10] and Sun et al. [8]. To ensure a comprehensive and systematic comparison, we also constructed several other prediction models based on the features selected in the literature [8,10], including the K-nearest neighbour (KNN), decision tree (DT), random forest (RF) and adaptive boosting (AdaBoost) methods. According to Table 2 and Table 3, it can be seen that on the independent test set that the performance of SecProCT was better than that of the other conventional machine learning methods.

To better evaluate the performance of our model, we also compared our model with the existing deep learning architecture on an independent test set. In recent years, there have been many excellent works applying deep learning methods to biological sequence analysis. For example, a convolutional neural network was used in DeepSig to detect signal peptides in proteins [17]. DanQ used a hybrid CNN and a bidirectional long-term short-term memory network to predict the characteristics and functions of DNA sequences [18]. In DeepLoc, an end-to-end model based on a convolutional neural network, bidirectional long short-term memory network and an attention mechanism was used to predict the protein subcellular localisation [19]. Du et al. used a novel end-to-end deep learning model based on multilane capsule network (CapsNet-SSP) with differently sized convolution kernels to identify saliva-secretory proteins [20]. To ensure a fair comparison with other deep learning architectures, we used the same balanced dataset and training strategy as the proposed SecProCT model to train these deep learning models.

Specifically, we used the deep learning architecture proposed in DeepSig, DanQ, DeepLoc and CapsNet-SSP to replace the network architecture part of the code. Table 4 and Table 5 describe the performance comparison results between different deep learning architectures for predicting blood-secretory proteins and saliva-secretory proteins. As shown in Table 4 and Table 5, the performance of SecProCT on the independent test set was significantly better than the other deep learning architectures.

In addition, we also compared our method with other methods on the iMSP test dataset [13]. Zhang et al. recently proposed a method for predicting secretory proteins in plasma based on discrete firefly optimisation, which had satisfactory results [14]. Since the authors did not provide the relevant source code and program, we evaluated the performance of our model on the iMSP test dataset and compared the experimental results with those in article [14]. Table 6 shows the performance metrics of SecProCT and these methods applied to the iMSP test dataset. Table 6 shows that the performance of SecProCT was better than that of the other methods.

### 2.2. Ablation Study on Binary Classification

To understand what makes our framework effective, we compared the performance of different combinations of convolutional (Conv) blocks, capsule network (CapsNet) blocks and transformer encoder (Trans) blocks using 10-fold cross-validation on the training dataset. For all combinations, the same training set and validation set were used. The performance comparison results of different combinations for predicting blood-secretory protein and saliva-secretory protein are shown in Table 7 and Table 8. The prediction results of all combinations on blood-secretory proteins and saliva-secretory proteins were satisfactory, with accuracies of 0.921 and 0.892 and MCCs of 0.686 and 0.759, respectively.

Comparing these results with the prediction results without the CapsNet block and without the Trans block (MCCs of 0.674, 0.722 and 0.647, 0.750, respectively), it can be found that the CapsNet block and Trans block can improve the prediction performance. In addition, by comparing the architecture using all blocks and without the Conv block, we can see that the Conv block also improved the prediction performance. As shown in Table 7 and Table 8, the average accuracy, sensitivity, specificity, MCC and AUC using all combinations for predicting blood-secretory protein and saliva-secretory protein were 0.921, 0.911, 0.931, 0.686, 0.950 and 0.892, 0.834, 0.950, 0.759, 0.920, respectively, which were better than those of the other architectures.

### 2.3. Evaluating the Performance of Human Secretory Protein Prediction

To further validate the effectiveness of the proposed model, we collected additional human secretory proteins that do not overlap with our training set. We collected known blood-secretory proteins from the published literature [21] and removed the proteins in the training set. Finally, 968 new blood-secretory proteins were obtained to evaluate the performance of the blood-secretory protein prediction. There are 3449 proteins that have been detected in saliva by using LC–MS/MS analyses in several published studies [22,23,24,25,26,27], among which 1628 have been detected in more than one study. Of these 1628 proteins, 529 are secretory proteins listed in the secretory protein database. Then, we removed the proteins in the training set from the 529 proteins and obtained 215 saliva-secretory proteins.

**Figure 1:**
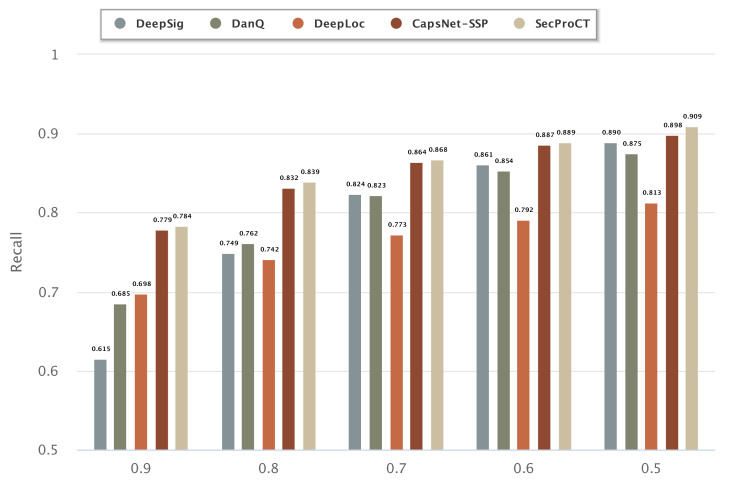
Prediction results of known blood-secretory proteins.

**Figure 2:**
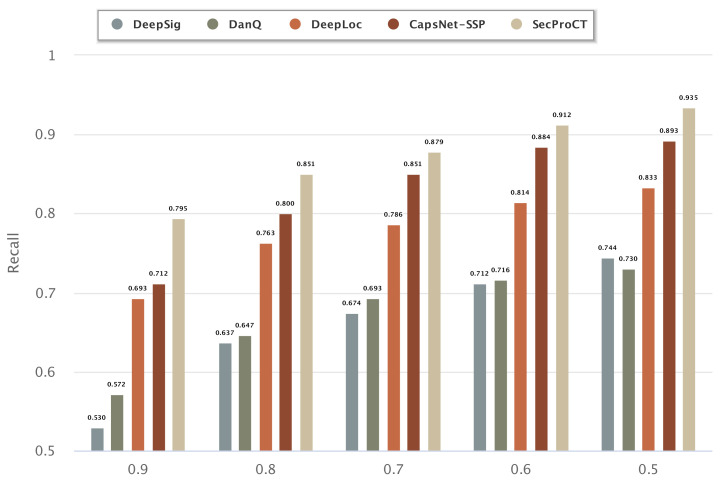
Prediction results of known saliva-secretory proteins.

Figure 1 and Figure 2 show the prediction results for the 968 blood-secretory proteins and 215 saliva-secretory proteins using the model based on DeepSig, DanQ, DeepLoc, CapsNet-SSP and SecProCT. Figure 1 and Figure 2 show the recall when using different threshold values in the prediction. As shown in Figure 1 and Figure 2, the prediction results of SecProCT are better than those of the other methods.

### 2.4. Evaluating the Performance of Cancer Biomarker Prediction

To further confirm the effectiveness of the proposed model in detecting disease markers, we also evaluated the prediction results of existing cancer biomarkers in blood and saliva. The existing blood biomarkers were collected from published research [28,29]. In this study [28], Ahn et al. analysed biomarkers in the blood of patients with colorectal cancer and obtained 41 biomarkers for the diagnosis of colorectal cancer. In study [29], Ahn et al. analysed the blood biomarkers of patients with small-cell lung cancer and obtained 106 biomarkers for the diagnosis of lung cancer. We combined the cancer markers of the above two studies and removed the overlapping proteins and finally obtained 129 cancer markers in blood. We also collected saliva in head and neck squamous cell carcinoma (HNSCC) [8], oral squamous cell carcinoma (OSCC) [30], lung cancer (LC) [31] and breast cancer (BC) [32] and obtained cancer markers in saliva.

**Figure 3:**
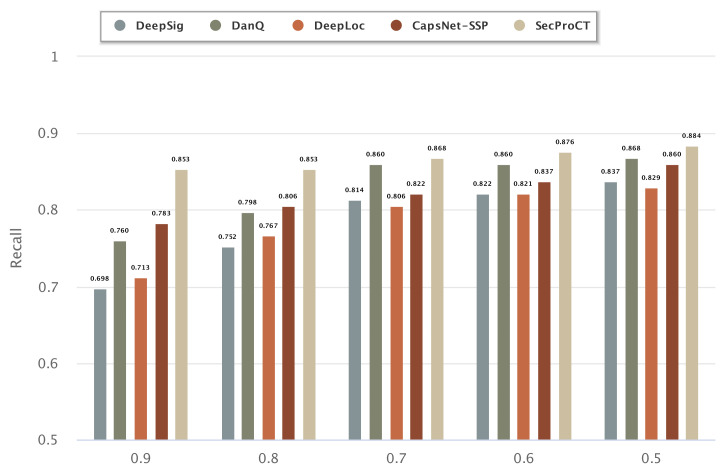
Prediction results of known cancer markers in blood.

**Figure 4:**
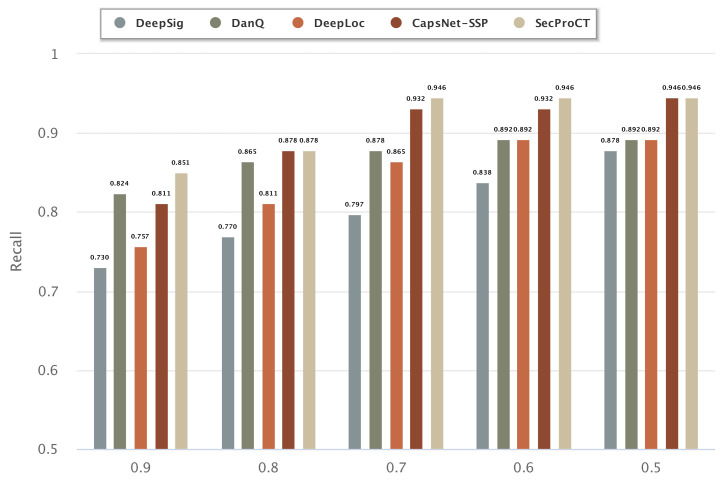
Prediction results of known cancer markers in saliva.

Figure 3 and Figure 4 show the prediction results of 129 cancer markers in blood and 215 cancer markers in saliva using the model based on DeepSig, DanQ, DeepLoc, CapsNet-SSP and SecProCT. Figure 3 and Figure 4 show the recall by using different threshold values in the prediction. As shown in Figure 3 and Figure 4, the prediction results of SecProCT are better than those of the other methods.

## 3. Discussion

The main contribution of this paper was the proposal of an end-to-end prediction model based on deep learning, which can accurately identify secretory proteins only using protein sequence information and obtain statistical significance for identifying existing cancer biomarkers in blood and saliva. Compared with traditional machine learning methods, our model can automatically learn feature representations and predict results from amino acid sequences, which can reduce feature incompleteness or bias that may result from feature engineering and the feature selection process of traditional machine learning methods. The prediction results of the proposed model are better than those of existing conventional machine learning methods and deep learning methods for biological sequence analysis. The disadvantage of the proposed model is that the training time is longer than that of traditional machine learning methods.

## 4. Materials and Methods

### 4.1. Data Collection

First, we collected human secretory proteins from the secreted protein database (SPD)  [33], the mammalian protein subcellular localisation database (LOCATE) [34], the Universal Protein Resource (UniProt) [35] and the Subcellular Proteome KnowledgeBase (MetazSecKB) [36]. At present, there are already many databases that have collected proteins that can be detected in blood and saliva. In addition, some studies have reported the proteins that appear in blood and saliva. We collected the detectable proteins in blood and saliva from the Sys-BodyFluid database [37], which contains proteins from different human tissues collected from many proteomics studies. We collected blood proteins from the Plasma Proteome Project (PPP) database [38] and other studies of blood proteins [10]. We also collected saliva proteins from two other saliva proteomics studies [24,39]. Then, the intersections of the human secretory proteins and the blood and saliva proteins were calculated to obtain the blood-secretory proteins and saliva-secretory proteins, respectively. In addition, to prevent learning bias due to protein redundancies, we removed the proteins that had a mutual sequence similarity above 30% using the CD-HIT tool [40]. Finally, 486 blood-secretory proteins and 450 saliva-secretory proteins remained, which were regarded as positive data. Among these blood-secretory proteins, 380 proteins were classified into the positive training set, and the other 106 proteins were classified into the positive test set. Among these saliva-secretory proteins, 350 proteins were classified into the positive training set, and the other 100 proteins were classified into the positive test set.

Since no protein is clearly defined as a non-blood-secretory protein and non-saliva-secretory protein in existing studies, generating a negative dataset was a difficult step in the data collection process. In this article, we used a method similar to that proposed by Cui et al. [10] to select proteins from the Pfam family that do not contain positive data, suggesting they are non-blood-secretory proteins and non-saliva-secretory proteins. To reduce the influence of protein families that only contain a small number of proteins, we only selected proteins from protein families that have more than ten proteins. For each protein family, we selected three members to construct the negative data. Then, we used the CD-HIT tool to remove the proteins with a mutual sequence similarity higher than 30% from the negative data. Among the 3900 obtained non-blood-secretory proteins, 3800 proteins were divided into the negative training set, and the other 100 proteins were divided into the negative test set. Among the 1850 non-saliva-secretory proteins, 1750 proteins were divided into the negative training set, and the other 100 proteins were divided into the negative test set.

### 4.2. Input Sequence Encoding

After completing the data collection, we used PSI-BLAST [15] to perform sequence alignment in the “Uniref50” database [16] to obtain evolutionary profile information for protein sequence encoding. Specifically, the profile of each protein is actually a normalised position-specific scoring matrix (PSSM) [41], which is generated based on the amino acid frequencies at every position of the multiple alignment using PROFILpro [42]. The sequence lengths of the different proteins are different, and the lengths of the obtained position-specific scoring matrices are also different. If the longest protein coding length is used uniformly, the overall training time of the model will be too long. Therefore, we appropriately cropped the encoded data and set 1000 as the maximum length of the input data. When the length of the PSSM of a certain protein was less than 1000, 0 was filled at the end of it. Since most of the information about blood-secretory proteins and saliva-secretory proteins is stored at the beginning (N-terminus) and the end (C-terminus) of the sequence [8,10], when the length of the PSSM of a certain protein exceeds 1000, we selected 500 amino acids from the beginning (N-terminus) of the protein and 500 amino acids from the end (C-terminus) of the protein to avoid losing the N-terminus and C-terminus classification signals. When applying this rule, only 11.74% and 16.32% of human blood-secretory proteins and saliva-secretory proteins are truncated.

### 4.3. Architecture Design

**Figure 5:**
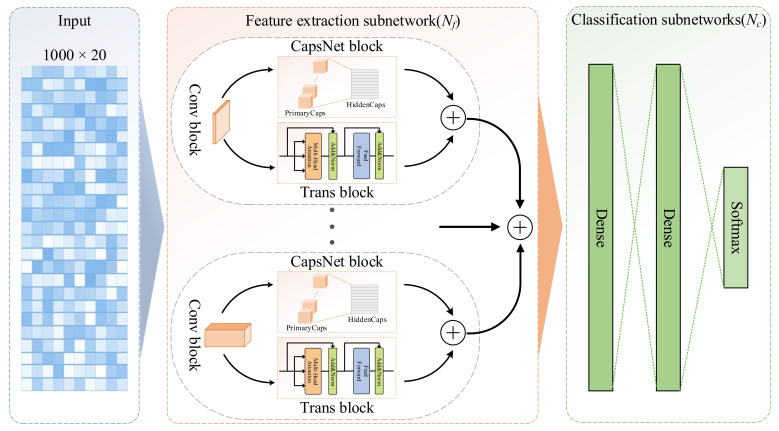
Architecture of the proposed model.

The prediction architecture of the proposed SecProCT model is shown in Figure 5. The model contains a feature extraction subnetwork ($N_{f}$) and a classification subnetwork ($N_{c}$). The input of the model is a 1000 × 20 profile of the PSSM for each protein. The feature extraction subnetwork ($N_{f}$) consists of multiple feature extraction blocks, each of which contains a convolutional (Conv) block, a capsule network (CapsNet) [43] block and a transformer encoder (Trans) [44] block, and one concatenation layer which is used to concatenate the extracted features. The classification subnetwork ($N_{c}$) contains two fully connected dense layers, and one softmax output layer. In this article, the feature extraction subnetwork ($N_{f}$) contains eight feature extraction blocks, which have different Conv layers containing 1D convolution kernels of a specified size with a stride of 1 and a rectified linear unit (ReLU) activation function [45].

The size of the kernel in the Conv layer determines the length of the motif that can be extracted from the protein sequence. In the network architecture proposed by Armenteros et al. for predicting protein subcellular localisation [19], the kernel size of each channel is 1, 3, 5, 9, 15, and 21. In this paper, to better identify the important characteristics of the signal peptides and transmembrane domains for predicting secretory proteins [10], we added kernel sizes of 27 and 33 to the network architecture. These Conv layers can convert the initial PSSM profile of each protein to intermediate-level features, which are then fed into the CapsNet block and the Trans block for further feature abstraction.

In each feature extraction block, the CapsNet block and Trans block are parallel connections. Each CapsNet block contains the PrimaryCaps layer and the HiddenCaps layer. The PrimaryCaps layer is also a 1D convolutional layer containing eight convolutional capsule channels. Each capsule contains 64 convolution units, and each capsule contains a 1D convolution kernel with a stride of 1. The squashing activation function is used in the PrimaryCaps layer to scale the lengths of the capsules to [0, 1] as follows:

(1) $$v_{j}\;=\;\frac{∥s_{j}∥}{1\;\;\;+\;\;\;{∥s_{j}∥}^{2}}\frac{s_{j}}{∥s_{j}∥},$$

where $s_{j}$ is the input vector of capsule *j* and $v_{j}$ is its output vector.

There are eight 16D capsules in the HiddenCaps layer, which can map the input protein to different states. The computation between the PrimaryCaps layer and HiddenCaps layer is the same as the calculation in the original CapsNet paper [43]. The structure of the Trans block is similar to the encoder part in the original Transformer paper [44], which contains two sub-layers. The first layer is a multi-head self-attention mechanism layer, and the second layer is a fully connected feed-forward network. For each of the two sub-layers, a residual connection is employed followed by layer normalisation. In the Trans block, the output dimension of all sub-layers and embedding layers is defined as 16 to facilitate these residual connections.

### 4.4. Model Training

For predictions based on deep learning frameworks, one of the challenging problems that must be solved is how to train highly accurate generalised models with small samples. If the number of training samples is much smaller than the number of parameters in the deep learning model, there is a high risk of overfitting during the training process [9]. Transfer learning refers to the transfer of knowledge learned in related tasks to new tasks [46], thereby improving the learning effect, which has been successfully applied to a deep learning framework for small sample problems [47]. To solve the problem of small samples in secretory protein prediction, we used the concept of transfer learning to fine-tune the prediction model of secretory proteins by the pre-trained prediction model of protein subcellular localisation. The dataset of protein subcellular localisation is from DeepLoc, which contains 13,858 proteins with ten main locations [19]. We trained the framework of the proposed model on the dataset of protein subcellular localisation and then transferred all layers except the output layer to the secretory protein prediction model. Finally, we used the blood-secretory protein and saliva-secretory protein data to fine-tune the entire model. At the same time, the bagging ensemble learning method [48] was used in the training process to reduce the impact of unbalanced data. By training the model on multiple selected balanced training subsets, we obtained multiple independent classifiers. Then, the final prediction result was calculated by averaging the results of these independent classifiers. The bagging ensemble learning algorithm used to train our proposed model is given below:
**Algorithm 1:** Bagging Algorithm [49]**Input:**
  $S^{+}$: Training set with positive samples   $S^{-}$: Training set with negative samples   *T*: Number of iterations
*n*: The size of a random selection   
I: Weak classifier
1:**for **t=1 to *T* **do**2:$S_{t}^{-}=$ Random Sample Selection $\left(n,S^{-}\right)$3:$h_{t}=I\left(S_{t}^{-}\cup S^{+}\right)$4:**end for**
**Output:**
  Bagged classifier:$H\left(x\right)=\mathrm{sgn}\left(\sum_{t=1}^{T}h_{t}\left(x\right)\right)$ where $h_{t}\in \left[-1,1\right]$


Here, $S^{+}$ contains 380 proteins and 350 proteins for blood and saliva, respectively. $S^{-}$ contains 3800 proteins and 1750 proteins for blood and saliva, respectively. The number of iterations *T* is 10, and the size of the random selection *n* is 380 and 350 for blood and saliva, respectively.

Dropout, early stopping and L2 regularisation strategies were used to prevent overfitting during the training of complex deep learning models. The dropout strategy prevents overfitting and optimises the generalisation ability of the model by adding multiple dropout layers to the prediction model. Another strategy to reduce overfitting was early stopping, that is, stopping training early at the right time during the training process. Specifically, when the loss of validation data does not decrease within a preset iteration period, the training process stops early [50]. The third strategy we used to prevent overfitting is to use regularisation in the training process of neural networks. The final optimisation function contains two items: one is a loss term used to quantify the degree of fitting the model to the data, and the other is a regular term used to quantify the complexity of the model and to prevent overfitting. In this study, we used L2 regularisation to prevent the overfitting of the deep learning model.

In the model training process, the proposed deep learning model and other deep learning models used the same training strategies. These deep learning models were optimised using the Adam stochastic optimisation method [51] and used the following parameters: the exponential decay rate of the first moment estimation was 0.9, the exponential decay rate of the second moment estimation was 0.999, and the learning rate was 0.0005. We used cross-entropy loss as the loss function to measure the difference between the true distribution and the predicted distribution of secretory proteins and non-secretory proteins. The proposed model and the comparative models used in the experiment were all executed on a workstation equipped with Ubuntu 18.04.2 LTS operating system, Intel Core i7-7800X CPU, 128 GB RAM and NVIDIA GeForce RTX 2080 Ti GPU. The software environment for our proposed deep learning model is Keras 2.2.4 and TensorFlow 1.13.1.

### 4.5. Performance Measurements

To compare the performance of different prediction models, the accuracy, sensitivity, specificity, Matthews correlation coefficient (MCC) and area under the receiver operating characteristic (ROC) curve (AUC) were used as performance metrics. The corresponding formulas are as follows:

(2) $$\mathrm{accuracy}=\frac{TP+TN}{N_{total}},$$

(3) $$\mathrm{sensitivity}\;=\;\mathrm{recall}\;=\;\frac{TP}{TP+FN},$$

(4) $$\mathrm{specificity}\;=\;\frac{TN}{TN+FP},$$

(5) $$\mathrm{MCC}\;=\;\frac{TP\times TN-FP\times FN}{\sqrt{\left(TP+FN)(TP+FP)(TN+FP)(TN+FN\right)}},$$

(6) $$\mathrm{AUC}\;=\;\frac{\sum_{p_{0}\in D^{0}}\sum_{p_{1}\in D^{1}}g(p_{0},p_{1})}{|D^{0}\left|\cdot \right|D^{1}|},\;\;\mathrm{where}\;g\left(p_{0},p_{1}\right)=\left\{\begin{array}{c} 1\;\;\;\;\mathrm{if}(f\left(p_{0}\right)<f\left(p_{1}\right))\\ 0\;\;\;\;\mathrm{otherwise}\; \end{array}\right.,$$

where TP represents the true positive, TN represents the true negative, FP represents the false positive, FN represents the false negative, and $N_{total}$ is the total number of samples in the validation or test set. $D^{0}$ and $D^{1}$ are the positive dataset and the negative dataset, respectively. *f*(.) represents the prediction function. In machine learning, MCC has a distribution range of [−1, 1], which is a measurement used to quantify the quality of the binary classification. When the value is 1, it means that the prediction is exactly the same as the label. When the value is −1, it means that the prediction result and the label are completely opposite. Since the accuracy, sensitivity, specificity and MCC depend on the threshold, it is necessary to choose a threshold to calculate their value. In the evaluation of binary classification, MCC produces more informative and truthful scores than accuracy and AUC. Therefore, in this article, the threshold is set where the MCC reaches its maximum value. AUC is one of the most important evaluation indexes to measure the performance of any classification model. It is a performance measurement of classification problems under different threshold settings.

## 5. Conclusions

In this study, we propose an end-to-end model based on capsule network and transformer architecture, which can accurately identify human secretory proteins from only amino acid sequence information. The first step in constructing the model was to use PSI-BLAST to transform the input protein sequence into a normalised position-specific scoring matrix (PSSM). In addition, to solve the problem of unbalanced datasets, the bagging ensemble learning method was applied during the training process. Then, the PSSM of the training set was fed into the model to train the model parameters. Finally, the trained model was used to predict the secretory proteins in the test set, and its performance was verified to be better than the existing methods. At the same time, our model can accurately detect known human secretory proteins and cancer biomarkers.

## Figures and Tables

**Table 1:** Existing studies for predicting secretory proteins.

Study	Body Fluid	Algorithm	Advantage	Disadvantage	Ref
Cui et al. (2008)	Blood	SVM	1	5	[10]
Liu et al. (2010)	Blood	Ranking	2	5	[11]
Wang et al. (2013)	Saliva	SVM	4	5	[12]
Sun et al. (2015)	Saliva	SVM	1	5	[8]
Zhang et al. (2018)	Body fluids	SVM	3	5	[13]
Zhang et al. (2019)	Blood	DFO *	1	5	[14]

1: High prediction accuracy; 2: Not need negative training dataset; 3: Strong biological correlation; 4: For all body fluids; 5: Need feature construction and feature selection. * DFO: Discrete firefly optimisation.

**Table 2:** Performance comparison with machine learning methods for predicting blood-secretory proteins.

Methods	Accuracy	Sensitivity	Specificity	MCC	AUC
KNN	0.879	0.848	0.911	0.759	0.873
Decision Tree	0.858	0.852	0.864	0.716	0.838
Random Forest	0.866	0.818	0.916	0.736	0.922
AdaBoost	0.859	0.838	0.881	0.719	0.912
SVM	0.893	0.877	0.910	0.787	0.925
SecProCT	0.917	0.906	0.930	0.835	0.967

**Table 3:** Performance comparison with machine learning methods for predicting saliva-secretory proteins.

Methods	Accuracy	Sensitivity	Specificity	MCC	AUC
KNN	0.778	0.649	0.907	0.575	0.809
Decision Tree	0.772	0.692	0.851	0.550	0.740
Random Forest	0.781	0.804	0.758	0.563	0.836
AdaBoost	0.792	0.703	0.881	0.593	0.847
SVM	0.781	0.784	0.778	0.562	0.857
SecProCT	0.909	0.898	0.919	0.817	0.956

**Table 4:** Performance comparison with deep learning architectures for predicting blood-secretory proteins.

Methods	Accuracy	Sensitivity	Specificity	MCC	AUC
DeepSig	0.893	0.906	0.880	0.786	0.939
DanQ	0.850	0.783	0.920	0.708	0.927
DeepLoc	0.898	0.877	0.920	0.797	0.940
CapsNet-SSP	0.893	0.897	0.890	0.786	0.942
SecProCT	0.917	0.906	0.930	0.835	0.967

**Table 5:** Performance comparison with deep learning architectures for predicting saliva-secretory proteins.

Methods	Accuracy	Sensitivity	Specificity	MCC	AUC
DeepSig	0.792	0.745	0.838	0.586	0.867
DanQ	0.802	0.745	0.859	0.608	0.886
DeepLoc	0.843	0.755	0.929	0.695	0.891
CapsNet-SSP	0.888	0.847	0.929	0.779	0.948
SecProCT	0.909	0.898	0.919	0.817	0.956

**Table 6:** Performance of SecProCT and other methods on the iMSP test set.

Methods	Accuracy	Sensitivity	Specificity	MCC	AUC
SecretomeP	0.762	0.632	0.787	0.340	0.764
SRTpred	0.782	0.678	0.802	0.392	0.770
iMSP-U	0.829	0.631	0.866	0.443	0.821
iMSP-H	0.850	0.538	0.908	0.441	0.817
SCRIP	0.858	0.716	0.884	0.537	0.865
SecProCT	0.873	0.809	0.884	0.596	0.920

**Table 7:** Performance of different architectures for predicting blood-secretory proteins on the training set.

Architectures	Accuracy	Sensitivity	Specificity	MCC	AUC
Without Conv block	0.882	0.853	0.912	0.604	0.915
Without CapsNet block	0.916	0.903	0.928	0.674	0.947
Without Trans block	0.905	0.889	0.921	0.647	0.938
All	0.921	0.911	0.931	0.686	0.950

**Table 8:** Performance of different architectures for predicting saliva-secretory proteins on the training set.

Architectures	Accuracy	Sensitivity	Specificity	MCC	AUC
Without Conv block	0.875	0.848	0.902	0.671	0.909
Without CapsNet block	0.891	0.857	0.925	0.722	0.918
Without Trans block	0.888	0.828	0.948	0.750	0.915
All	0.892	0.834	0.950	0.759	0.920

## Data Availability

The pipeline of the model, prediction data and results can be accessed at the following URL: http://www.csbg-jlu.info/SecProCT/, accessed on 15 August 2021.
